# Microplastic Pollution: An Emerging Threat to Terrestrial Plants and Insights into Its Remediation Strategies

**DOI:** 10.3390/plants11030340

**Published:** 2022-01-27

**Authors:** Arpna Kumari, Vishnu D. Rajput, Saglara S. Mandzhieva, Sneh Rajput, Tatiana Minkina, Rajanbir Kaur, Svetlana Sushkova, Poonam Kumari, Anuj Ranjan, Valery P. Kalinitchenko, Alexey P. Glinushkin

**Affiliations:** 1 Academy of Biology and Biotechnology, Southern Federal University, 344006 Rostov-on-Don, Russia; msaglara@mail.ru (S.S.M.); tminkina@mail.ru (T.M.); terra_rossa@mail.ru (S.S.); randzhan@sfedu.ru (A.R.); 2 Department of Botanical and Environmental Sciences, Guru Nanak Dev University, Amritsar 143005, India; snehrajput89@gmail.com (S.R.); rajanbir19@yahoo.com (R.K.); 3 Department of Biosciences, Himachal Pradesh University, Shimla 171005, India; poonamkumari0989@gmail.com; 4 All-Russia Research Institute for Phytopathology RAS, 5 Institute St., Big Vyazyomy, 143050 Moscow, Russia; kalinitch@mail.ru (V.P.K.); glinale1@mail.ru (A.P.G.); 5 Institute of Fertility of Soils of South Russia, Krivoshlykova St., Persianovka, 346493 Moscow, Russia

**Keywords:** plastic pollution, higher plants, morpho-physiological responses, reclamation techniques

## Abstract

Microplastics (MPs) are ubiquitous and constitute a global hazard to the environment because of their robustness, resilience, and long-term presence in the ecosystem. For now, the majority of research has primarily focused on marine and freshwater ecosystems, with just a small amount of attention towards the terrestrial ecosystems. Although terrestrial ecosystems are recognized as the origins and routes for MPs to reach the sea, there is a paucity of knowledge about these ecological compartments, which is necessary for conducting effective ecological risk assessments. Moreover, because of their high persistence and widespread usage in agriculture, agribusiness, and allied sectors, the presence of MPs in arable soils is undoubtedly an undeniable and severe concern. Consequently, in the recent decade, the potential risk of MPs in food production, as well as their impact on plant growth and development, has received a great deal of interest. Thus, a thorough understanding of the fate and risks MPs, as well as prospective removal procedures for safe and viable agricultural operations in real-world circumstances, are urgently needed. Therefore, the current review is proposed to highlight the potential sources and interactions of MPs with agroecosystems and plants, along with their remediation strategies.

## 1. Introduction

There are countless advantages of using plastics, including chemical and mechanical qualities, but the pollution caused by the extensive use of plastic has been a major concern in recent years. As a result of the inappropriate disposal of plastic garbage, it is now known to contaminate the environment, with simultaneous impacts on living beings [1]. A considerable amount of MPs (i.e., plastic particles less than 5 mm in diameter) has been found on every continent, from the highest mountain peaks to the deepest ocean depths [2]. Moreover, the concern regarding the omnipresence of MPs has been arising since the study conducted in 2004 [3] that revealed the presence of MPs in most of the samples collected from different beaches. The contamination of seawater is a continued problem, as one million metric tons of plastic is reported to be dumped into the ocean [4]. Likewise, MPs occurrence in terrestrial ecosystems is increasing but have been investigated less extensively than MPs in marine environments. Thus, the pollution caused by the excessive use of plastic film mulch and a low recycling rate are serious concerns [5]. Thus, plastic pollution is expected to worsen in the foreseeable future.

MPs originate from a range of sources, including both primary and secondary MPs. Primary MPs are any plastic fragments or particles that are already less than 5.0 mm in diameter when they enter the environment, such as microbeads, microfibers, and plastic pellets. They are extensively employed in the cosmetics and pharmaceutical industries and are produced specifically to be small [6,7]. Whereas secondary MPs are produced when larger plastics are fragmented by external forces, including sunlight, wind, and water, as well as chemical, biological, and mechanical factors [8]. Now, coming to the emission of primary and secondary MPs, it is documented that both are released directly from shipping, fishing, and coastal activities, along with other sources, such as untreated sewage and treated effluents from industrial and municipal wastewater treatment plants, etc. [9].

Owing to the extensive use of plastic, MPs are pervasive, including in the atmosphere, soils, seawater, and freshwater, as well as in the sediments of Artic Lake [10]. Apart from the contamination caused by their widespread use, MPs have been reported to adsorb a variety of inorganic and organic pollutants, due to their small volume and high specific surface area, resulting in the accumulation of pollutants, posing a slew of risks to the surrounding flora and fauna, including humans [11]. Further, they can interact with a vast spectrum of biota because of their long lifetime, widespread distribution across habitats, and small size; yet evidence on MP exposure and the associated repercussions is still sparse in the literature. Therefore, many researchers have explored the impacts of MPs on a variety of marine and freshwater organisms over the last decade, but the situation is reversed in the case of terrestrial plants. However, plants are key primary producers in terrestrial ecosystems and are also equally as vital to the environment as other organisms, and therefore, they should be carefully investigated as part of MP research. With this context in mind, we designed this comprehensive review to provide a holistic view of research developments on the exposure-mediated effects of MPs on plants, their sources, recent trends in MP pollution, uptake, phytotoxicity, and remediation approaches.

## 2. Sources of MPs

**Figure 1:**
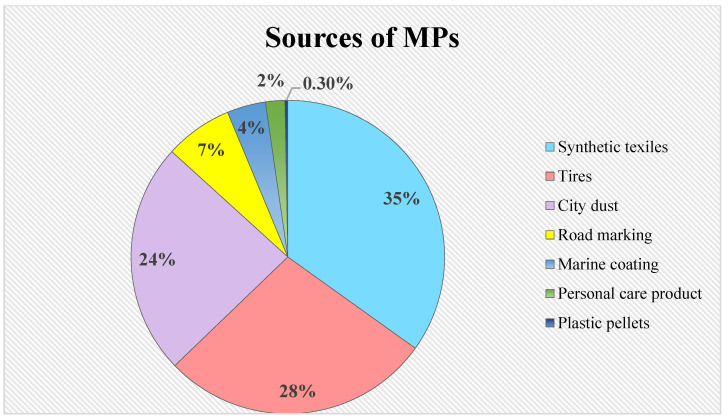
Potential sources of microplastics to contaminate environments (Source: International Union for Conservation of Nature, 2017).

As plastic manufacturing and consumption continue to grow, despite certain regulations, the situation will exacerbate, increasing the likelihood of plastic becoming more widespread. The reported potential sources of MPs are synthetic textile, tires, personal care products, etc. (Figure 1). Thus, a piling of plastic trash in the environment is the result of the material’s long shelf life, unsustainable use, and disposal [12]. Furthermore, the additives and hazardous compounds in the plastics are released throughout the decomposition process [13]. These compounds are impervious to environmental degradation and can easily accumulate in soil and water. It is anticipated that 500 million tons of plastic waste will be produced by 2050 [14].

Furthermore, if current trends continue, the ocean may contain more plastic than fish by 2050 (https://www.unep.org/interactive/beat-plastic-pollution/; accessed on 20 December 2021). Besides, over 300 million tons of MPs are expected to be produced, which will continue to contaminate agroecosystems as either primary or secondary MPs [15]. In the environment, MPs are the diverse array of particles with varying sizes, shapes, chemical compositions, and specific densities that come from a range of sources [16]. MPs generated and released to the environment in the micro-size range are known as primary MPs, whereas secondary MPs come from the fragmentation of larger plastic components [17].

**Figure 2:**
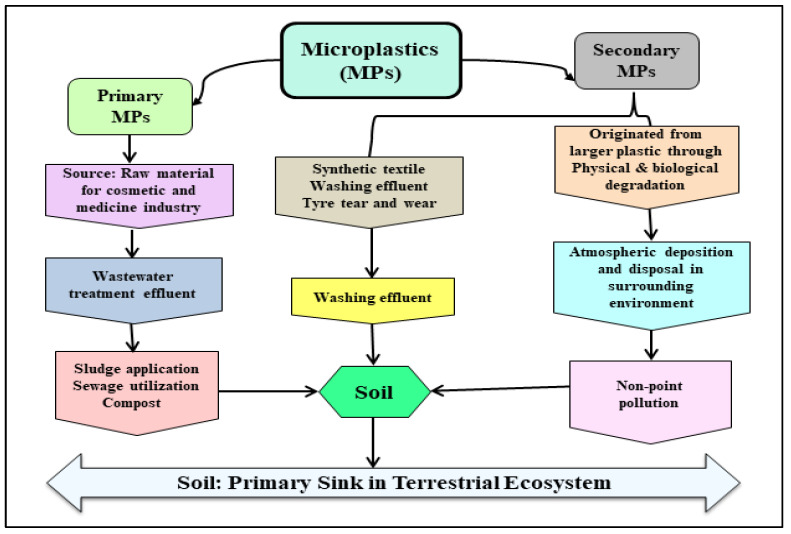
Schematic representation of the types of microplastics, sources, and sinks in the terrestrial ecosystem.

Plastic mulch films, greenhouse materials, and soil conditioners are examples of direct sources in agriculture. Contrarily, the indirect sources of MPs are common littering, irrigation with reclaimed water, and the application of biosolids [18]. More details on the origin and fate in the environment of primary and secondary MPs are summarized in Figure 2.

## 3. Interactions of MPs with Agroecosystems and Plants

### 3.1. MPs in Agroecosystems

**Figure 3:**
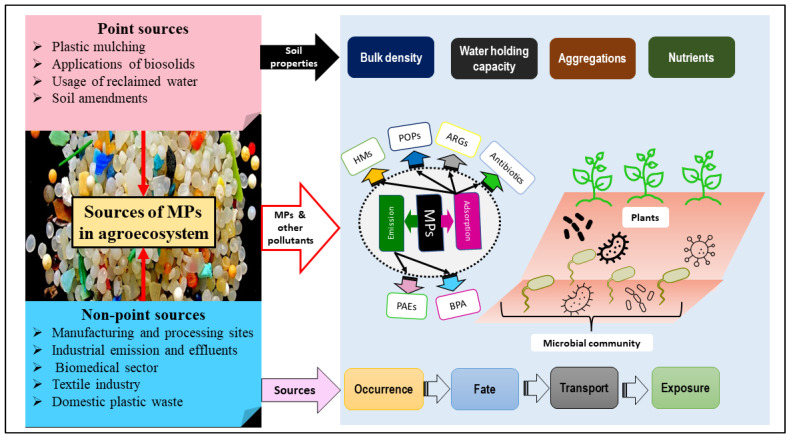
Representation of microplastics sources in the agroecosystems, the impacts on soil properties, and their fate; HM: heavy metal, POPs: persistent organic pollutants, ARG: antibiotics and bacterial/phage resistance genes, PAEs: phthalic acid esters, BPA: bisphenol-A.

In this section, we will discuss those plastics which end up in agroecosystems. In a study, the annual and maximum plastic loadings in agroecosystems were estimated using available data and estimates for Europe, the United States, and Australia. According to the observational data, up to 2.5 million tons of MPs are predicted to end up in the oceans each year, with two-thirds of that amount attributable to the synthetic fibers released during washing and tire attrition when driving [19]. Besides, it is believed that approximately 95% of MPs that pass via sewage treatment plants are absorbed into biosolids. Thus, after the applications of biosolids for fertilization purposes, MPs obtain their routes to the agroecosystems (Figure 3). Further, it has also been observed that reclaimed water is utilized for irrigation in various parts of the world, making it another significant source of MP pollution [20]. Besides biosolids, in agroecosystems, composts made from unsorted domestic garbage or mixed municipal solid waste, as well as source-separated garden organic waste, are sources of plastic contamination [21]. Thus, these factors, as well as abrasion and fragmentation caused by mixing and transit, contribute to the physical degradation of plastics. In this respect, some studies indicated that biosolids contributed significantly to MPs pollution in agroecosystems, i.e., up to 430,000 tons (Europe) and 300,000 tons (North America) [22,23].

MPs are reported to be further fragmented or weathered by solar ultraviolet radiation, as well as by increased oxygen availability and temperature. Afterward, fragmented MPs migrate vertically through the soil profile and horizontally along the surface of soils, resulting in the spread of plastic contamination over a wide range of habitats, including deep soil, groundwater, aquatic environments, etc. [24]. Besides, MPs are well-known for their long-term persistence in soils. In this context, some studies demonstrated that after burying the plastics in the forms of pro-oxidant mulching films in soil for 8.5 years and when analyzing the degradation of LDPE film in laboratory conditions for 10 years there was no degradation, because all the plastics were recovered in their original forms [25]. Likewise, in another study, the biodegradation after 32 years was recorded for different polymers that were buried in soil, and the results revealed that there was no degradation at all [26]. Thus, the problem is quite concerning, because plastics are known to deteriorate in 20 to 500 years, depending on the substance, structure, and environmental conditions. However, in the case of MPs, the precise information is not available regarding their specific fate and the time required for complete degradation.

MPs are recorded to modify soil characteristics, such as bulk density, water-holding capacity, and soil structures. Depending on the type of MPs, the qualities of the soil are affected to varying degrees [27]. The other major concern has been raised by the omnipresence of MPs i.e., in the soil, MPs can combine with other pollutants (organic pollutants, heavy metals, antibiotics, etc.) to negatively impact their inherent organisms [28]. In addition, the interaction of MPs with organic and inorganic contaminants can alter the behavior of these contaminants, and MPs can serve as a significant conduit for the migration of these contaminants throughout the subsurface [29,30,31,32]. In a study, MP fibers were reported to lower soil bulk density and promote soil aeration, which reduced root penetration resistance and increased root growth [33]. In another study, after exposing MPs to earthworms, adverse impacts were observed [34], which might be due to its impact on soil porosity and water content, which eventually suppressed plant growth and development.

Thus, these modulations in soil structure caused by MPs pollution may have an impact on microbial composition and functions. MPs exposure has direct and indirect effects on the food chain, due to their ubiquity, size, source volume, chemical components, and complicated interactions with biotic and abiotic factors in the agroecosystems [35].

### 3.2. Mechanism of MP Uptake in Plants

In the last decade, plant scientists have started to investigate the mechanisms of MP uptake and translocation in plants. MPs have been demonstrated to permeate seeds, roots, stems, leaves, fruits, and plant cells, but only to a certain extent and depending on their size and type [36]. Commonly, it is assumed that plants are unlikely to be able to absorb MPs because of their high molecular weight and large size, which prevents them from penetrating cellulose-rich plant cell walls. However, their uptake has been reported by some reports when they are broken down to their nanoforms [37,38]. Thus, nanoplastics (NPs) can find their way to enter the plant cells. Further, some engineered nanomaterials (ENMs), including metals, oxides, and carbon allotropes, have been shown to enter plants via roots and scatter in various plant tissues, implying a significant likelihood of plant uptake of NPs [37].

Likewise, in a study, after the exposure of polystyrene NPs (20 nm) to rice, a substantial distribution of PS was found in the roots’ intercellular spaces [27]. Moreover, nano-polystyrene (50 nm) was found in different compartments of the primary roots of onion after exposure for 72 h, reflecting that nano-polystyrene is capable of penetrating a variety of biological barriers and, finally, entering root cells [39]. Similarly, polystyrene-NPs (0.2 µm) were found to be absorbed by the roots of lettuce and wheat that were also recorded to be transported into shoots. Thus, due to alterations in the cellular membranes and biochemistry, the accumulating NPs had a detrimental effect on crop health [38]. Hence, to ensure safe food production, further research is needed on the interactions of MPs and NPs, as well as their fate in the agroecosystems.

### 3.3. MPs and Plants

After the pursual of literature, it was found that exposure of MPs has been documented to impact the morpho-physiological traits of plants directly or indirectly. However, the impacts vary greatly under the exposure of micro- or nano-plastics, depending on plant type and plastic features (Table 1). The details of the MP-mediated consequences on germination, growth, and the biochemical indices of plants are discussed below.

#### 3.3.1. Germination and Growth

Germination is a critical stage in the life cycle of plants, and it begins with the absorption of water, which includes establishing the metabolic reactions required for seed germination [40]. In the case of micro (nano) plastics, a reduction in seed germination rates was observed in *Lepidium sativum* L. [41]. Similarly, the exposure of different particle sizes (2 nm and 80 nm) and various concentrations (0, 10, 50, 100, and 500 mg/L) of polystyrene MPs to herbaceous plants (*Trifolium repens*, *Orychophragmus violaceus*, and *Impatiens balsamina*) caused a reduction in germination rates [42]. The germination indices, such as germination percentage, germination vigor, and the germination index, of rice seedlings were recorded to be inhibited under the stress of polystyrene MPs only at higher concentrations (1000 mg/L) [43].

The influence of NPs and MPs on the growth of terrestrial plants has received less investigation. Soils with MPs-containing sludge promoted tomato plant growth while delaying and reducing fruit output. However, the authors stated that more research is needed to confirm these findings and clarify the mechanisms of MPs’ potential effects on plants [44]. In the study of Qi et al. [18], the effects of low-density polyethylene (LDPE) and starch-based plastic (biodegradable) MP films of different sizes on wheat grown in pots were recorded. The MP films were reported to considerably influence the wheat growth at the vegetative and reproductive stages. Moreover, the biodegradable plastic mulch films had a more significant impact on wheat development than polyethylene. In another work, six different MPs were reported to adversely impact the plant biomass, tissue elemental composition, root traits, and soil microbial activities in *Allium fistulosum* [45].

#### 3.3.2. Biochemical and Physiological Responses

Crops (plants) are vital for human survival and ecological health. However, there are several abiotic and biotic stressors, including MPs, that decrease the economic outcomes of edible plants. According to a substantial number of studies, MPs have been found to have both negative and positive effects on plants’ performances [46,47]. Further, MPs not only impact the soil properties, soil fauna, and microbes but also interfere with the metabolic activities of plants via their uptake and accumulation. In plants, the accumulation of MPs in their organs and the adherence to the surfaces of the roots or seeds has been linked with the decrement in the water and nutrient uptake [47,48]. To date, regarding the effects of MPs on plants, some researchers believe that the size of MPs is a crucial cue that is primarily responsible for the extent of phytotoxicity [49]. However, others claim that the shape of the MPs is critical for exhibiting the morpho-physiological implications in plants [18,50].

Until now, when it comes to metabolic and physiological consequences, it has been found after a thorough review of the literature that most reports are limited to aquatic plants. Moreover, only a few studies have been found that are primarily concerned with terrestrial plants. Therefore, a summary of such studies that measured the implications of MP exposure on the terrestrial plants is given in Table 1.

**Table 1:** Effects of MPs on the morpho-physiological parameters of different plants.

MP(s), Size, and Concentrations	Plant(s)	Germination, Growth, and Phytotoxic or Phyto-Stimulating Responses	References
Polypropylene (PP), Polyethylene (PE), polyvinylchloride (PVC), and polyethylene terephthalate (PET); 40–50 μm; 0.02%, 0.1, and 0.2% (*w*/*w*)	*Cucurbita pepo* L.	All MPs impaired root and, particularly, shoot growth. All MPs reduced the leaf size, pigment content, and photosynthetic efficiency. Moreover, all MPs changed the micro- and macro-elemental profiles. PVC was found to be the most toxic among all MPs, and PE was found to be less toxic.	[51]
Polystyrene (PS) -MPs and polytetrafluoroethylene (PTFE); with sizes of 0.1–1 μm (S) and 10–100 μm (L); 0%, 0.25%, and 0.5%	*Oryza sativa* L.	Both PSMP and PTFE lowered the relative abundance of Geobacteria and Anaeromyxobacter while inhibiting root activity. PSMP and PTFE also reduced the hemoglobin content, which subsequently retarded the rice growth. The activities of soluble starch synthase and pyrophosphorylase in rice grains were reduced by PSMP and PTFE, and, thus, starch accumulation decreased.	[52]
Micro-sized fluorescently labeled PS; 1 µm; 10 mg/mL	Indica rice variety Xiuzhan-15	PS-MPs were detected in different organs of rice seedlings. Moreover, PS-MP microspheres were found to be accumulated in the vascular networks of plants. Thus, the study confirmed the translocation of PS-MPs to the aboveground parts of the crop.	[48]
PS-NPs; 93.6 nm; 0, 0.1, and 1 mg/L	*Lactuca sativa* L.	PS-NPs significantly decreased the morphological and growth indices of lettuce compared to the control. Declines were observed in the pigment content and the activities of antioxidative enzymes. Ps-NPs induced a significant enhancement in the rate of electrolyte leakage rate. PS-NP exposure also resulted in substantial reductions in micronutrients and critical amino acids.	[53]
PE-MPs; 6.5 and 13 µm; 0, 10, 50, 100, 200, and 500 mg/L	*Glycine max* and *Vigna radiata*	Dry weight and root length were reduced by PE-MPs in soybean, while in mung bean it increased the root length. The exposure of PE-MPs to soybeans reduced germination associated parameters, i.e., energy, the germination index, and the vigor index.	[54]
PS-MPs; 100 nm (PS-1) and 1 μm (PS-2); 0, 0.1, 1, and 10 mg/L	*Oryza sativa* L.	PS-1 and PS-2 elevated root length, root surface area, and the number of root tips, but they lowered main root length in a dose-dependent manner. Both PS-MPs significantly increased the number of root tips. PS-1 m was shown to be more phytotoxic than PS100 nm.	[55]
High-density poly ethylene (HDPE), low-density poly ethylene LDPE, PP, PET; 0.31–2.11 mm; 17,870–47,130 particles/kg of dry soil.	*Lycopersicon esculentum* Mill.	Micro(nano)plastics at a low concentration enhanced plant growth. High concentrations of MPs resulted in a reduction in plant biomass. Moreoever, plant biomass was found to be lowered when MP concentrations were high.	[44]
PE-MPs; 0.5%, 1%, 2%, 5%, and 8% *w*/*w*; 200–250 μm	*Triticum aestivum*	PE-MPs adversely impacted the biomass and length of roots and shoots in a dose-dependent manner. PE-MPs at the 1% level were found to stimulate root elongation. The activities of antioxidative enzymes were increased at 0.5 to 5% concentrations of PE-MPs, while they were reversed at 8%. PE-MPs disrupted the functioning of the photosynthetic system of wheat leaves.	[56]
PS; 5.64 ± 0.07 µm; 2 g/mL	*Hordeum vulgare*	In contrast to control plants, plants stressed by PS had significantly higher concentrations of H_2_O_2_ and O^2−^ in their roots. PS-MPs disturbed the cellular homeostasis and the antioxidative defense system of plants via exerting modulatory impacts on roots and shoots. However, the alteration trends were alike in roots and shoots Moreover, PS-PMs significantly altered the concentrations of the different phytohormones compared to the control.	[57]
PP, PE, PVC, and a commercial mixture (PE + PVC); 0.02% (*w*/*w*)	*Lepidium sativum*	All MPs exhibited significant impacts on the germination, morphobiometric parameters, and oxidative stress bioindicators. PVC was recorded as being more toxic than the other MPs	[58]
PVC with different particle sizes: PVC-a (100 nm to 18 μm) and PVC-b (18 to 150 μm); 0.5, 1, and 2%	*Lactuca sativa* L.	PVC-a and PVC-b showed no significant effect on root activity. Increases in the total length, surface area, volume, and diameter of roots were observed. PVC-a at 1% concentration significantly increased SOD activity. PVC-a improved carotenoid synthesis but was inhibited by PVC-b.	[59]
PS; 20 and 190 nm; 0.01–1.0 g/L	*Allium cepa* L.	Root length was found to decrease with increasing concentrations of PS. PS exposure caused cytological abnormalities, as well as genotoxicity. Moreover, PS-mediated stress caused oxidative stress in the plants.	[39]
PET, PP, PE, and PVC; 5− 3000 μm; 0.02% (*w*/*w*).	*Lepidium sativum* L.	Seed germination percentage, plants’ morphological parameters, and total biomass were found to be decreased. Long-term exposure prompted oxidative damage by altering the contents of H_2_O_2_, glutathione, and ascorbic acid in plants. Plant responses to different polymers were recorded to be varied considerably. PVC was found to the more toxic than other plastics.	[60]
PS-MPs; 5 mm (PS-1) and 100 nm (PS-2); 10, 50, and 100 mg/L	*Vicia faba*	Biomass and the CAT activity of roots decreased due to PS-1, while POD activity significantly increased. PS-2 significantly decreased growth, only at 100 mg/L. Experimental data from the micronucleus test and antioxidative enzyme activities reflected that PS-2 mediated a higher level of genotoxic and oxidative stress than PS-1.	[61]
LDPE and biodegradable plastic; 0.05–7 mm; 10 g/kg	*Triticum aestivum*	Wheat plants’ vegetative and productive growth were both inhibited by MP exposure. In addition, plants‘ biomass was decreased by LDPE and biodegradable plastic.	[18]

In the case of plants’ exposure to MPs, there are two major concerns: whether plants can absorb and accumulate MPs and whether MPs have an impact on plant growth and the quality of food produced.

**Figure 4:**
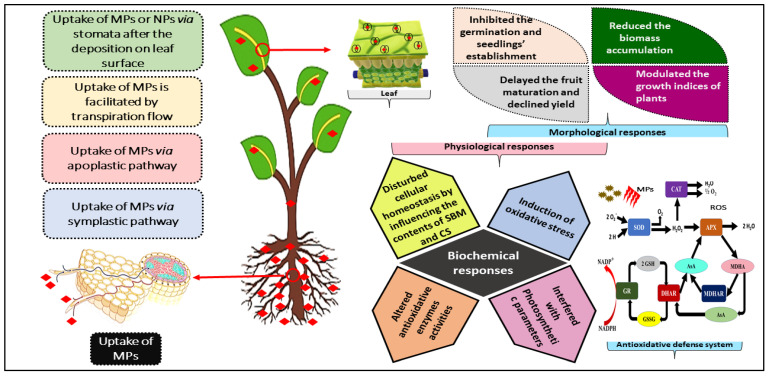
Scheme of microplastic uptake and morpho-physiological implications in plants; SOD: superoxide dismutase, POD: peroxidases, APX: ascorbate peroxidase, MDHA: monodehydroascorbate, MDHAR: monodehydroascorbate reductase, AsA: ascorbate, GR: glutathione reductase, GSH: oxidized glutathione, GSSG: glutathione reductase, NADP^+^: nicotinamide adenine dinucleotide phosphate (oxidized form), NADPH: nicotinamide adenine dinucleotide phosphate (reduced form).

In this context, from the studies summarized in Table 1, it is demonstrated that MPs can accumulate in plants, which is directly related to altered cellular homeostasis, which eventually impacts the plant economic outcomes and raises food security concerns. The detailed scheme of the uptake and morpho-physiological implications in plants is depicted in Figure 4.

## 4. Remediation Strategies of MPs

The chemical additives included in plastics, such as phthalates, bisphenol A, and polybrominated diphenyl ethers, have the potential to cause hazardous consequences on plants and even in other living organisms, including humans, when ingested [13,62]. Furthermore, MPs have a high adsorption capacity, which renders them susceptible to transporting a variety of pollutants [63]. Hence, in this section, the potential methods to remove MPs from the environments are described.

### 4.1. Techniques for Biodegradation

#### 4.1.1. Hyperthermophilic Composting (hTC) Technology

Recently, due to the prevalence of hyperthermophilic bacteria, a hTC has been developed that operates at temperatures greater than 90 °C [64]. Moreover, hTC is performed at temperatures 20–30 °C higher than traditional thermophilic composting (cTC), leading in more rapid bioconversion, better maturity, and a shorter composting period [64]. A study demonstrated the successful utilization of hTC technology for the *in-situ* biodegradation of sludge-based MPs [65]. The high temperature used in this removal strategy transforms the large-sized MPs into smaller ones which further facilitates their biodegradation. The magnified activity of hyperthermophilic bacteria ultimately raises the temperature during hTC, which, in turn, is expected to support the thermolytic cleavage of the −C−C− bonds. The most common bacteria discovered using high-throughput sequencing were *Thermus* sp., *Bacillus* sp., and *Geobacillus* sp., which efficiently executed MP biodegradation during hTC [65]. Thus, hyperthermophilic bacteria play a crucial role in MPs biodegradation during hTC, revealing a possible technique for removing sludge-based MPs from the physical world. Moreover, there is room for more research into using hTC to remove MPs from environmental matrices.

#### 4.1.2. Whole-Cell Biocatalysis 

With the course of scientific advancement, researchers came up with the idea of utilizing whole microorganisms, such as bacteria, yeasts, and filamentous fungi, to act as whole-cell biocatalysts, on the basis of their immobilization potential. Moreover, these whole-cell biocatalysts can display different functional proteins of interest on the surface [66]. Recently, a novel method of combined processing, based on whole-cell biocatalysts of alkali and organisms for the efficient biodegradation of PET was reported [67]. The bacterial strain used in this study was the engineered strain F5, which was procured by evolutionary engineering. The strain can grow with PET particles under alkaline conditions (pH = 11), using it as a sole carbon source. Additionally, the strain F5 was improved further to make it an alkali-tolerant bacterium, *Comamonas testosterone* F6. Further, the micro-size particles of PET were utilized as the substrate to simulate the MPs biodegradation. In the bacterial whole-cell biodegradation method, the products do not accumulate in the culture medium and are used by the strain for growth, thus avoiding the feedback inhibition of products. Moreover, PET degradation based on whole-cell biocatalysis has significant advantages over free enzymes in terms of decreasing the time and cost of the protein purification process [68].

#### 4.1.3. Periphytic Biofilm

In this MP removal technique, periphytic biofilm was used for the biodegradation of three MPs, namely PP, PE, and PET [69]. Different carbon sources alone and in combination were used in this study, such as glucose, peptone, and their mixture. The results of the study revealed that the addition of glucose augments the biodegradation rate of all three MPs by periphyton biofilm, while peptone, and glucose and peptone together have inhibitory effects. Furthermore, MiSeq sequencing unveiled that the microbial community structure was affected by the different carbon sources. The dominant phyla in the natural biofilms were *Deinococcus-thermus*, *Bacteroidetes* sp., *Proteobacteria* sp., and *Cyanobacteria* sp., and the addition of glucose increased their relative abundance. In another study, the use of periphyton and epixylon biofilms was referred as an efficient and ecofriendly technique for the biodegradation of PE [70].

### 4.2. Microorganism-Mediated Biodegradation

MPs represent a unique ecological niche for microbes that offers them support for growth and colonization, while also acting as a carbon source. The biodegradation of MPs comprises three consecutive steps, i.e., biodeterioration, biofragmentation, and assimilation. For investigating the biodegradation of MPs, numerous microbes were isolated from different environmental sources that can degrade MPs.

#### 4.2.1. Bacteria

Bacteria are found almost everywhere, mostly in soil, water, and the environment. Many bacterial species are characterized by the ability to breakdown contaminants in the environment [71]. Numerous studies on the bacteria-mediated degradation of MPs have focused on the use of pure bacterial cultures, bacterial consortium, and bacterial biofilms under laboratory conditions. These cultures were isolated from different sources, such as sediment, sludge, wastewater, soil, and the marine environment by enrichment culturing [72]. In a study, Park and Kim (2019) used mesophilic mixed bacterial culture isolates, predominantly *Bacillus* sp. and *Paenibacillus* sp. procured from a municipal landfill site for the biodegradation of micro-sized polyethylene (40 µm to 600 µm) [73]. The results revealed that the dry weight of particles was reduced to 14.7% and the mean particle diameter was reduced to 22.8% after 60 days, as observed by field-emission scanning electron microscope. In a similar study, Auta et al. (2018) reported the biodegradation of polypropylene using *Rhodococcus* sp. strain 36 and *Bacillus* sp. strain 27, isolated from mangrove sediments [74]. The study unveiled that both bacterial strains were able to utilize polypropylene for growth and the reduction in the polymer mass was 6.4% by *Rhodococcus* sp. and 4.0% by *Bacillus* sp. after incubating for 40 days.

#### 4.2.2. Fungi

The ability of the fungus *Zalerion maritimum* in the biological decomposition of polyethylene was evaluated [75]. In a study from 12 different eco-geographical locations along the west coast of India, a total of 109 fungal isolates were recorded. After the analysis based on morphological factors and molecular tools, *Aspergillus terreus* strain MANGF1/WL and *Aspergillus sydowii* strain PNPF15/TS were the most efficient polythene deteriorating fungi [76]. In a study, the degradation of HDPE film by two marine fungi, namely *A. tubingensis* VRKPT1 and *A. flavus* VRKPT2, without any pre-treatment and pro-oxidant additives was observed. Among both strains, the colonization, biofilm formation, and biodegradation of HDPE film were found higher by *A. flavus* VRKPT2 than *A. tubingensis* VRKPT1. Moreover, the authors stated that the fungal strains can degrade HDPE under in-vitro conditions. Therefore, they also give a viable solution to the HDPE polymer’s environmental danger [77].

#### 4.2.3. Algae

The effectiveness of cyanobacteria to cleanup MPs or NPs in nature has yet to be considered on priority. In a study, *Scenedesmus dimorphus*, *Anabaena spiroides*, and *Navicula pupula* were reported as the most prevalent species on the polyethylene bags that were collected from the suburban water bodies. Moreover, their effects on PE degradation were explored. The proliferation of microalgae was higher in the case of LDPE than that of HDPE, and *Anabaena spiroides* showed maximal PE degradation compared to the other algae [78]. Interestingly, microalgae can break down plastics via the formation of toxins or enzymes, as well as utilizing plastic polymers as carbon sources. However, algae-based degradation research is still in its early stages and is unlikely to be commercialized on a large scale. The progress of technology and the continuous R&D in bioplastics is critical [79].

### 4.3. Microbial Enzymes

Recently, the degradation of plastics via microbial enzymes has emerged as a viable method of depolymerizing waste petro-plastics for recycling or mineralizing [80]. In this method of biodegradation of plastics, the excretion of extracellular enzymes by the microorganism is reported as the first step, followed by the attachment of enzymes to the surface of the plastic. After the interaction of the enzyme with plastics, hydrolysis to short polymer intermediates takes place, and they are ultimately assimilated by microbial cells as a carbon source to release CO_2_ [80,81]. The commonly employed microbial enzymes employed in the biodegradation of MPs are laccase, esterase, hydrolase, lipase, carboxylesterase, cutinase, protease, etc.

Thus, multiple approaches for the removal of MPs from the environment have been developed and applied. While each method has its own set of advantages and disadvantages, the lack of sufficient literature on various MPs removal procedures makes it impossible to recommend a single method as the best available option. Moreover, it is possible to further investigate the various microorganisms and methods that have been well-established for the removal of other contaminants from the environmental media, which can be used in conjunction with one another [82,83].

## 5. Conclusions and Future Perspective

The exploration of so many facets of the MP life cycle remain a significant problem, despite the fact that MPs research began decades ago, and scientific knowledge has evolved significantly in recent years. In this work, we introduced some new implications of MPs’ interactions with higher plants. Such interactions, particularly vascular plants’ uptake and accumulation of MPs can have a variety of ecological effects in terrestrial ecosystems. First and foremost, it can result in the transfer of MPs at the different trophic levels. Thus, the contamination of MPs results in two major concerns, i.e., food security risks and persistence in different environmental matrices. So far, scientists have not dealt with a pollution problem as intricate and fraught with ambiguity as MPs research. Therefore, only via interdisciplinary collaboration among scientists can tackle such a complicated problem effectively and efficiently. Moreover, there must be a global perspective taken into consideration when dealing with MPs pollution, and it must not be restricted to the ocean and its influence on marine species. It must examine all conceivable ecosystems, with all of the biodiversity that they contain and that may interact with MPs. Besides, the studies on the screening of effective microbial strains, as well as other improved removal strategies, are urgently required in order to reduce the hazards generated by MPs in the environment to an acceptable level.

## Data Availability

Not Applicable.
